# Retraction Note to: Epigenetic regulation of HGF/Met receptor axis is critical for the outgrowth of bone metastasis from breast carcinoma

**DOI:** 10.1038/s41419-022-04992-6

**Published:** 2022-06-09

**Authors:** Paola Bendinelli, Paola Maroni, Emanuela Matteucci, Maria Alfonsina Desiderio

**Affiliations:** 1 Dipartimento di Scienze Biomediche per la Salute, Molecular Pathology Laboratory, Università degli Studi di Milano, Via L. Mangiagalli 31, Milano, 20133 Italy; 2 Istituto Ortopedico Galeazzi IRCCS, Via R. Galeazzi 4, Milano, 20161 Italy

Retraction to: *Cell Death and Disease* (2017) 8:e2578–e2578; 10.1038/cddis.2016.403, published online 02 February 2017

The Editors are retracting this article because of concerns with a number of figures. An investigation by the University of Milan confirmed that:

- The same vinculin bands for lanes 2–11 in Fig. 3b are used for vinculin lanes 1–10 in Fig. 5b, but the lysates are claimed to derive from different cell types.
- Vinvulin bands in Fig. 3d had been used to represent vinculin in a different experiment published in [1].
- In Fig. 4a, vinculin band in lane 2 is the same as vinculin band lane 2 in Fig. 4b; in Fig. 4a, the vinculin band in lane 7 is the same as that in lane 9 but it has been stretched horizontally; in Fig. 4b, vinculin bands in lanes 4 and 6 are the same.
- In Fig. 5b, vinculin bands in lanes 2, 3, & 4 are the same as those in lanes 8, 9, & 10.
- In Fig. 4b, vinculin bands are the same as those that appear representing different experiments in other publications namely [2–4].
- The vinculin bands in Fig. 5b had previously been used to represent those of a different experiment published in [5].
- Vinculin bands in Fig. 6b were used to represent vinculin in a different experiment published in Fig. 9b in [6].

The Editors therefore no longer have confidence in the data.

Maria Alfonsina Desiderio disagrees with this retraction. Paola Bendinelli, Paola Maroni and Emanuela Matteucci have not responded to any correspondence from the editor/publisher about this retraction.
